# Differential Plasma MMP-3 Levels in H1N1 and COVID-19 Pneumonia

**DOI:** 10.3390/diagnostics16213488

**Published:** 2026-10-25

**Authors:** Dooyoung Choi, Jamie Le, Shravani Etrouth, Sultan Almuntashiri, Gustavo Ramírez, Nora Regino, Luis Jiménez-Alvarez, Alfredo Cruz-Lagunas, Tatiana S. Rodriguez-Reyna, Joaquin Zuñiga, Xiaoyun Wang, Yin Zhu, Duo Zhang

**Affiliations:** 1 Department of Clinical and Administrative Pharmacy, College of Pharmacy, University of Georgia, Augusta, GA 30912, USA; 2 Department of Clinical Pharmacy, College of Pharmacy, University of Hail, Hail 55473, Saudi Arabia; 3 Laboratory of Immunobiology and Genetics, Instituto Nacional de Enfermedades Respiratorias Ismael Cosío Villegas, Tlalpan 4502, Mexico City 14080, Mexico; 4 Tecnologico de Monterrey, School of Medicine and Health Sciences, Mexico City 14380, Mexico; 5 Department of Immunology and Rheumatology, Instituto Nacional de Ciencias Médicas y Nutrición Salvador Zubirán, Tlalpan, Mexico City 14080, Mexico; 6 Pulmonary, Critical Care and Sleep Medicine, Department of Medicine, Baylor College of Medicine, Houston, TX 77030, USA; 7 Department of Medicine, Medical College of Georgia, Augusta University, Augusta, GA 30912, USA

**Keywords:** matrix metalloproteinase-3, SARS-CoV-2, influenza A virus, acute lung injury, acute respiratory distress syndrome, pneumonias

## Abstract

**Background:** Matrix metalloproteinase-3 (MMP-3) participates in extracellular matrix remodeling and lung injury, yet its behavior across different viral pneumonias remains unclear. We compared circulating MMP-3 levels in patients with coronavirus disease 2019 (COVID-19) and H1N1 pneumonia and evaluated their relationships with clinical severity. **Methods:** This retrospective study included hospitalized patients with laboratory-confirmed COVID-19 or H1N1 infection. Plasma MMP-3 concentrations were measured using enzyme-linked immunosorbent assay (ELISA). Clinical severity was assessed using the arterial oxygen partial pressure to fractional inspired oxygen ratio (PaO_2_/FiO_2_), Sequential Organ Failure Assessment (SOFA) score, Acute Physiology and Chronic Health Evaluation II (APACHE II) score, and hospital length of stay. Between-group comparisons and correlation analyses were performed among 109 patients, including 57 with H1N1 and 52 with COVID-19. **Results:** Plasma MMP-3 concentrations were higher in patients with H1N1 than COVID-19. Receiver operating characteristic analysis demonstrated excellent discriminatory ability of MMP-3 for differentiating H1N1 from COVID-19 pneumonia with an optimal cutoff value of 4.601 ng/mL. Higher MMP-3 levels were associated with lower bicarbonate levels in both cohorts, although the association reached statistical significance only in patients with H1N1. **Conclusions:** The circulating MMP-3 differs substantially between H1N1 and COVID-19 pneumonia and is substantially higher in H1N1 patients.

## 1. Introduction

Viral pneumonia caused by severe acute respiratory syndrome coronavirus 2 (SARS-CoV-2) and influenza A/H1N1 can produce diffuse injury to the alveolar–capillary barrier, leading to early exudation, surfactant dysfunction, and a rapid decline in lung compliance with worsening hypoxemia [1,2,3]. Both SARS-CoV-2 and H1N1 are important causes of acute lung injury and share similar clinical presentations, although their inflammatory profiles can differ. In these infections, heightened inflammatory responses and dysregulated protease activity further accelerate barrier disruption in patients who experience severe respiratory deterioration [4,5]. These observations highlight the variable severity of viral pneumonia and the importance of identifying indicators that reflect clinical deterioration. Reliable early biomarkers that reflect lung injury severity remain limited in viral pneumonia.

Matrix metalloproteinase-3 (MMP-3), or stromelysin-1, is a secreted enzyme that participates in extracellular-matrix remodeling and inflammatory tissue responses [6,7]. Elevated MMP-3 levels have been documented early in acute viral pneumonias, including coronavirus disease 2019 (COVID-19), and higher concentrations have been linked with more severe clinical presentations [8,9]. Observations from these settings have led to interest in MMP-3 as a measurable biomarker that may reflect early disease burden in viral respiratory infections. Given these findings, further characterization of circulating MMP-3 levels across different viral pneumonias may help clarify its clinical associations.

In this study, we examined circulating MMP-3 levels in patients with H1N1 or COVID-19 pneumonia and evaluated whether these concentrations differed by viral etiology. We also assessed how MMP-3 levels related to clinical severity indicators, including oxygenation status and established scoring systems. This approach was intended to clarify whether MMP-3 reflects acute disease burden in these two viral pneumonias.

## 2. Materials and Methods

### 2.1. Human Plasma Specimens and Ethics Approval

This retrospective study used previously collected, de-identified plasma specimens and was classified as non-human subjects research. Plasma from hospitalized COVID-19 patients was collected between March and October 2020, and plasma from H1N1 influenza patients was processed as described previously [10]. Samples were collected at the emergency departments of the Instituto Nacional de Enfermedades Respiratorias Ismael Cosío Villegas (INER) and the Instituto Nacional de Ciencias Médicas y Nutrición Salvador Zubirán (INCMNSZ), both in Mexico City. COVID-19 diagnosis was confirmed by real-time RT-PCR from respiratory specimens, and H1N1 infection was identified using rapid influenza diagnostic testing followed by molecular subtyping by RT-PCR, as previously reported [4]. Peripheral blood was drawn in the emergency department, processed to isolate plasma, and stored at ultralow temperatures until analysis [4]. At admission, illness severity was evaluated using the sequential organ failure assessment (SOFA) and acute physiology and chronic health evaluation II (APACHE II) scoring systems, along with measurement of the partial pressure of arterial oxygen to fraction of inspired oxygen ratio (PaO_2_/FiO_2_).

Ethics approval was obtained from the institutional review boards (IRB) of both participating clinical centers (INCMNSZ: IRB project number 3349 approved on 28 May 2020; INER: IRB project number B28-16 approved on 23 November 2016, and IRB project number B09-20 approved on 25 March 2020). Approval for biomarker analysis was also granted by the Augusta University Institutional Review Board (IRB 2070085). All procedures followed institutional guidelines and the Declaration of Helsinki.

### 2.2. Enzyme-Linked Immunosorbent Assay (ELISA)

Plasma samples were stored at −80 °C before analysis. MMP-3 concentrations were measured using a human MMP-3 ELISA kit (R&D Systems, Catalog # DY513, Minneapolis, MN, USA) according to the manufacturer’s instructions. Plasma samples were diluted prior to measurement and run in duplicate. Absorbance at 450 nm was recorded using the SpectraMax M5 Microplate Reader (Molecular Devices, Sunnyvale, CA, USA), and MMP-3 concentrations were calculated from a standard curve. Samples with MMP-3 concentrations below the assay’s limit of detection were excluded from the analysis. Of the collected samples, seven were excluded because MMP-3 concentrations were below the assay’s limit of detection.

### 2.3. Statistical Analysis

Data analysis was performed using IBM SPSS Statistics Version 30.0, and figures were generated with GraphPad Prism Version 10.5.0. Statistical significance was defined as *p* < 0.05. Demographic and clinical data were summarized, and correlations between MMP-3 levels and clinical variables were assessed. Normality was evaluated with the Shapiro–Wilk test. For normally distributed variables, results are presented as mean and standard deviation; for non-normally distributed data, median and interquartile ranges were reported. Group comparisons were conducted using Student’s *t*-test or the Mann–Whitney U test, as appropriate. Associations between plasma MMP-3 concentrations and clinical variables were evaluated using Spearman’s rank correlation coefficient. Multivariable regression analyses were performed to evaluate the independent association between plasma MMP-3 concentrations and clinical severity measures after adjustment for viral etiology (COVID-19 versus H1N1). Separate regression models were constructed for each clinical outcome.

## 3. Results

### 3.1. Study Population

The demographic, baseline characteristics, comorbidities, and outcomes of the patients stratified between COVID-19 and H1N1 populations are shown in Table 1. There were no significant differences in age or gender distribution between COVID-19 and H1N1 patients (*p* = 0.176 and *p* = 0.505, respectively). Smoking history was significantly more common among H1N1 patients than among COVID-19 patients (35.1% vs. 13.5%, *p* = 0.017). H1N1 patients had longer hospital stays and more frequently required mechanical ventilation (both *p* < 0.001). No significant differences were observed in mortality, diabetes mellitus, COPD, cancer, tuberculosis, obstructive sleep apnea or systemic arterial hypertension. Autoimmune disorders were more prevalent in the COVID-19 cohort (30.8% vs. 1.8%, *p* < 0.001). Blood samples were collected upon hospital admission and the interval from symptom onset to hospital admission was similar between groups. The circulating MMP-3 concentrations were significantly higher in H1N1 patients than in COVID-19 patients (6.45 ± 1.35 vs. 3.98 ± 0.75 ng/mL, *p* < 0.001).

### 3.2. MMP-3 Levels by Survival Status and Viral Etiology

**Figure 1:**
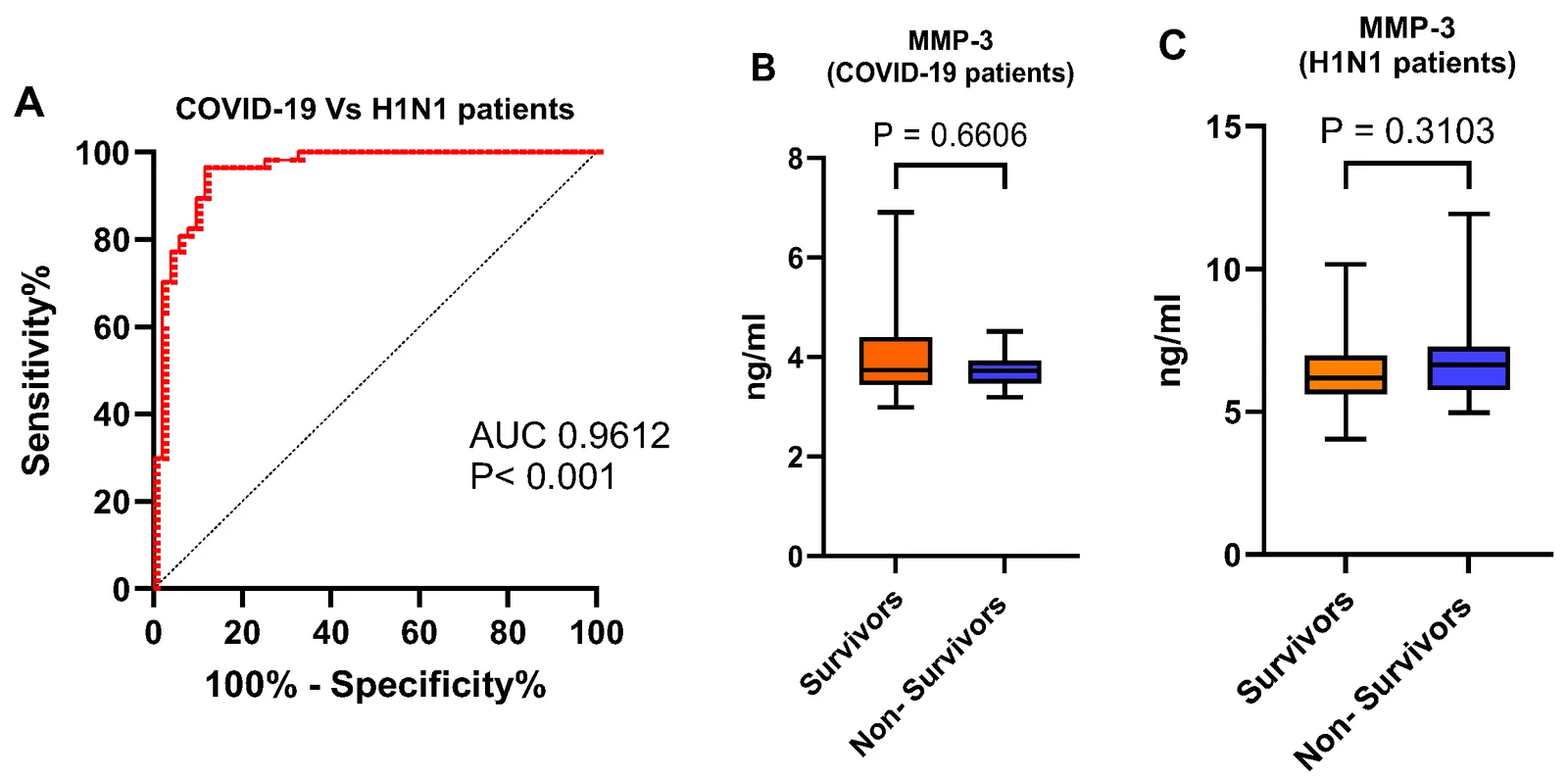
Plasma MMP-3 concentrations by survival status and viral etiology. (**A**) Receiver operating characteristic curve evaluating MMP-3 for differentiating H1N1 (*n* = 57) from COVID-19 (*n* = 52). (**B**) MMP-3 levels in survivors (*n* = 44) and non-survivors (*n* = 8) within the COVID-19 subgroup. (**C**) MMP-3 levels in survivors (*n* = 46) with non-survivors (*n* = 11) within the H1N1 subgroup.

To evaluate the ability of MMP-3 to distinguish between H1N1 and COVID-19 infections, an AUROC analysis was performed. MMP-3 demonstrated excellent discriminatory performance, with AUC of 0.961 (*p* < 0.001; 95% CI: 0.921–0.990). The optimal cutoff was 4.601 ng/mL, providing a sensitivity of 96.49%, a specificity of 88.46% and a Youden Index of 0.8495 (Figure 1A). The values above and below 4.6019 ng/mL are considered high and low MMP-3. Within each viral group, MMP-3 concentrations were similar between survivors and non-survivors. Among COVID-19 patients, median levels were 3.746 vs. 3.73 ng/mL (*p* = 0.6606) (Figure 1B). Among H1N1 patients, median levels were 6.18 vs. 6.66 ng/mL (*p* = 0.3103) (Figure 1C).

### 3.3. Clinical Characteristics According to MMP-3 Levels

The viral etiology, baseline characteristics, and outcomes of patients by MMP-3 levels are shown in Table 2. There were no significant differences in age distribution between low MMP-3 and high MMP-3 groups (*p* = 0.786). However, significant differences were observed in disease etiology, with COVID-19 patients predominantly in the low MMP-3 group (95.9% vs. 8.3%, *p* < 0.001) and H1N1 patients predominantly in the high MMP-3 group (91.7% vs. 4.1%, *p* < 0.001). Patients in the high MMP-3 group demonstrated significantly worse clinical outcomes across multiple parameters, including lower PaO_2_/FiO_2_ ratios (188 vs. 101.5, *p* <0.001), higher APACHE II scores (9 vs. 6.5, *p* = 0.01), higher SOFA scores (2 vs. 6, *p* < 0.001), and longer hospital length of stay (11 vs. 24 days, *p* = 0.001). Mortality rates (16.3% vs. 18.3%, *p* = 0.806) and bicarbonate levels (22.4 vs. 22.5, *p* = 0.798) were comparable between groups.

### 3.4. Correlation of MMP-3 with Clinical Parameters in COVID-19 Patients

**Figure 2:**
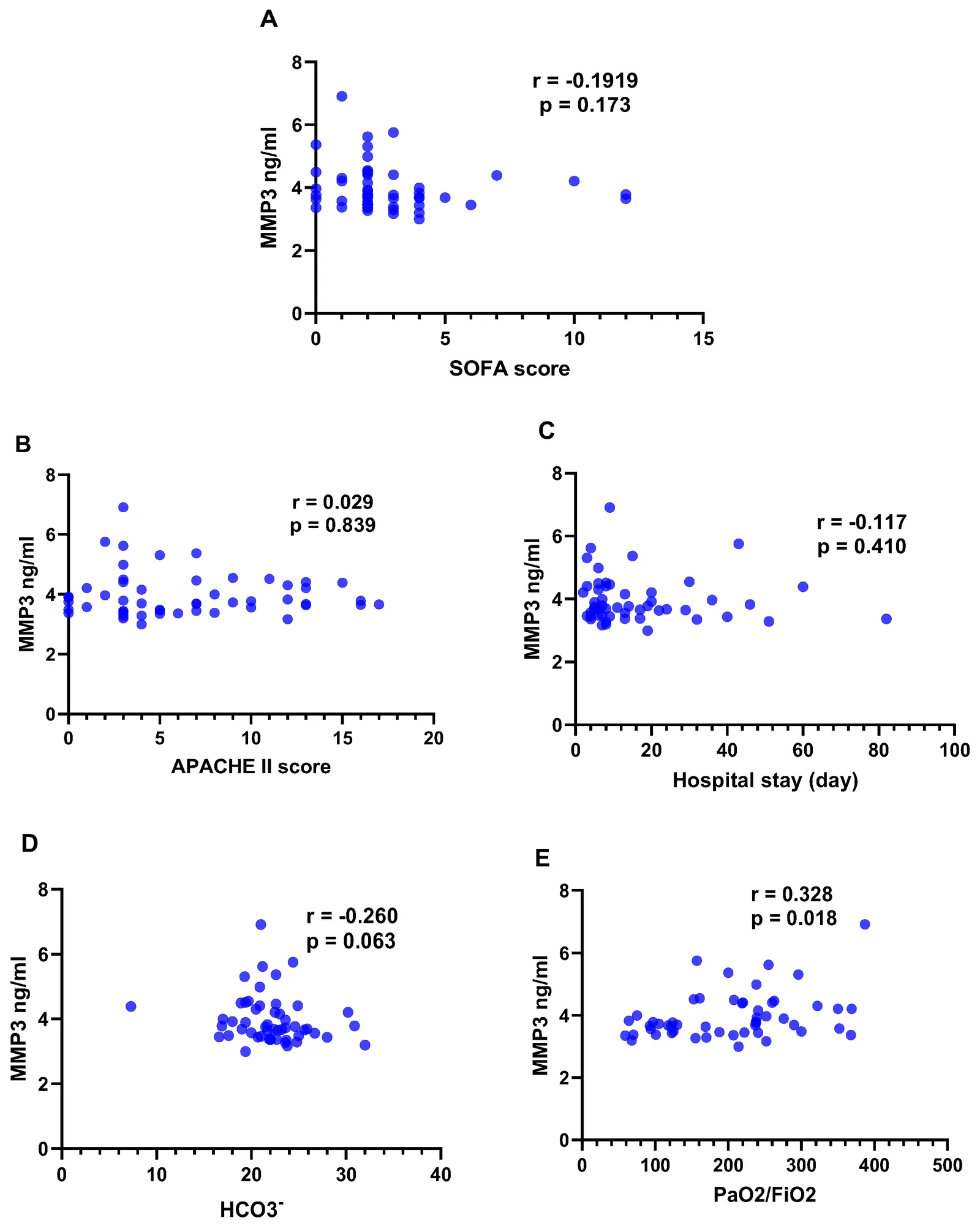
Correlation between plasma MMP-3 concentrations and clinical parameters in COVID-19 patients. (**A**) Relationship between MMP-3 and SOFA score. (**B**) Relationship between MMP-3 and APACHE II score. (**C**) Relationship between MMP-3 and Hospital length of stay. (**D**) Relationship between MMP-3 and HCO_3_^−^ levels. (**E**) Relationship between MMP-3 and PaO_2_/FiO_2_ ratio.

Next, we assessed whether MMP-3 levels were related to parameters of clinical severity in COVID-19 patients. No significant correlations were observed between MMP-3 levels and SOFA score (r = −0.1919, *p* = 0.173), APACHE II score (r = 0.029, *p* = 0.839), duration of hospital stay (r = −0.117, *p* = 0.410) or serum bicarbonate concentration (r = −0.260, *p* = 0.063) (Figure 2A–D). Although HCO_3_ demonstrated a weak inverse relationship with MMP-3, this did not reach statistical significance. MMP-3 showed significant positive correlation with PaO_2_/FiO_2_ ratios with MMP-3 concentration (r = 0.328, *p* = 0.018) (Figure 2E).

### 3.5. Correlation of MMP-3 with Clinical Parameters in H1N1 Patients

**Figure 3:**
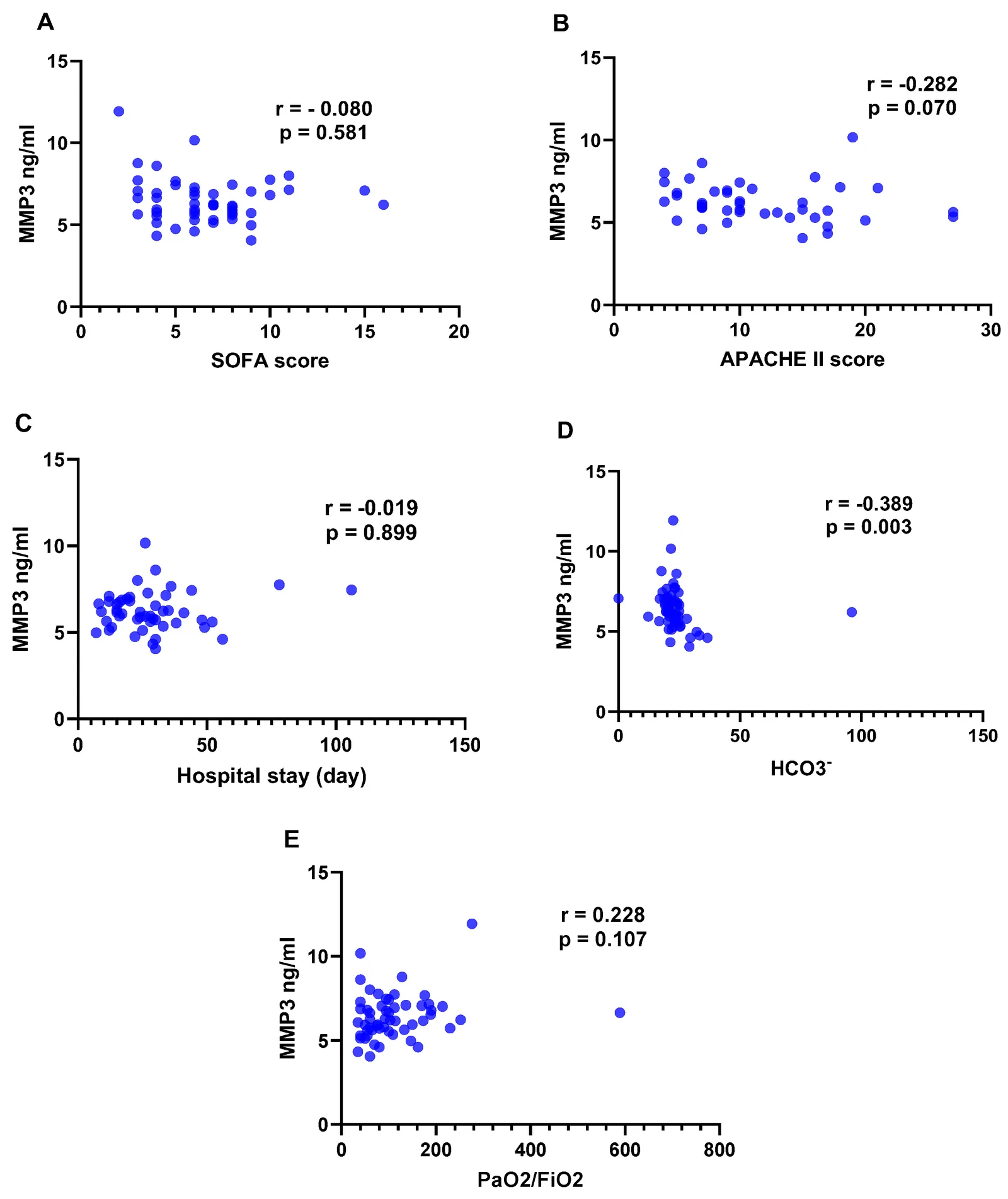
Correlation between plasma MMP-3 concentrations and clinical parameters in H1N1 patients. (**A**) Relationship between MMP-3 and SOFA score. (**B**) Relationship between MMP-3 and APACHE II score. (**C**) Relationship between MMP-3 and Hospital length of stay. (**D**) Relationship between MMP-3 and HCO_3_^−^ levels. (**E**) Relationship between MMP-3 and PaO_2_/FiO_2_ ratio.

We next assessed whether MMP-3 levels were related to parameters of clinical severity in H1N1 patients. No significant correlations were observed between MMP-3 levels and SOFA score (r = −0.080, *p* = 0.581), APACHE II score (r = −0.282, *p* = 0.070), duration of hospital stay (r = −0.019, *p* = 0.899) (Figure 3A–C) or PaO_2_/FiO_2_ ratio (r = 0.228, *p* = 0.107) (Figure 3E). Although the APACHE II score showed a weak negative correlation, the association did not reach statistical significance. MMP-3 levels demonstrated a significant negative correlation with bicarbonate levels (r = −0.389, *p* = 0.003), indicating that higher MMP-3 concentrations were associated with lower HCO_3_ values (Figure 3D).

### 3.6. Multivariable Analysis of the Association Between MMP-3 and Disease Severity Adjusting for Viral Etiology

To determine whether MMP-3 was independently associated with disease severity, multivariable linear regression analyses were performed with viral etiology (COVID-19 versus H1N1) included as a covariate (Table 3). After adjustment for viral etiology, MMP-3 was not independently associated with SOFA score (β = −0.334, *p* = 0.169), APACHE II score (β = −0.700, *p* = 0.221) or hospital length of stay (β = 0.558, *p* = 0.757). However, MMP-3 remained independently associated with PaO_2_/FiO_2_ ratio (β = 19.71, *p* = 0.012). Viral etiology remained a significant predictor of disease severity across multiple models, indicating that differences between COVID-19 and H1N1 contribute substantially to the observed clinical associations.

## 4. Discussion

In this study of patients with viral pneumonia, we compared circulating MMP-3 levels between H1N1 and COVID-19 infections and evaluated their relationship with clinical severity. We found that patients with H1N1 had markedly higher MMP-3 concentrations than those with COVID-19, and MMP-3 showed excellent ability to distinguish between the two viral etiologies. Across the full cohort, elevated MMP-3 levels were associated with worse clinical characteristics in the overall cohort; however, these associations may partly reflect differences in viral etiology because MMP-3 levels strongly segregated between H1N1 and COVID-19 patients. Together, these findings indicate that MMP-3 varies substantially by viral etiology and aligns with several markers of acute disease burden in viral pneumonia.

Several studies have evaluated circulating MMP-3 in COVID-19 and consistently report higher levels in patients with more severe illness. In a cohort of hospitalized patients spanning different World Health Organization (WHO) severity stages, Gelzo et al. found that serum MMP-3 was elevated at admission and showed a clear stepwise increase across severity categories [8]. In another study, Shi et al. similarly reported higher MMP-3 concentrations in COVID-19 patients compared with noninfected controls, along with positive correlations between MMP-3 and proinflammatory cytokines such as Interleukin-1β (IL-1β) and Interleukin-6 (IL-6) [9]. Our results are consistent with these observations, showing that MMP-3 is elevated in viral pneumonia and aligns with indicators of clinical severity.

Evidence on MMP-3 in H1N1 influenza mainly comes from experimental work. Feng et al. showed that MMP-3 has broad antiviral activity against H1N1 and that loss of MMP-3 impairs antiviral responses in vivo [7]. Beyond this mechanistic work, immunological studies show that H1N1 influenza is associated with a strong innate inflammatory response and elevated markers of tissue injury [4]. However, to date, there are no clinical studies describing circulating MMP-3 levels in patients with H1N1 pneumonia. Our findings, therefore, appear to be the first to report plasma MMP-3 concentrations in this population, showing substantially higher levels in H1N1 patients.

In our cohort, MMP-3 levels were substantially higher in H1N1 than in COVID-19, which may reflect the more intense early inflammatory response seen in influenza-related pneumonia. In a direct comparison of the two infections, pandemic H1N1 was associated with greater epithelial damage and a more intense innate cytokine profile, while COVID-19 showed a more mixed T-helper 1 (Th1)/Th2 pattern and different inflammatory signatures [4]. Experimental work also shows that MMP-3 is strongly induced during H1N1 infection and contributes to antiviral responses, whereas its upregulation during SARS-CoV-2 infection appears more modest [7]. Taken together, these findings provide a biological explanation for the substantially higher MMP-3 concentrations observed in our H1N1 patients compared with those with COVID-19.

Although MMP-3 contributes to endothelial dysfunction and vascular permeability, circulating MMP-3 levels may not solely reflect endothelial injury [11]. Influenza-induced pulmonary epithelial destruction and extracellular matrix remodeling may represent stronger drivers of systemic MMP-3 release than endothelial activation alone. Therefore, despite the more prominent vascular pathology observed in COVID-19, greater epithelial injury and matrix turnover during H1N1 infection may contribute to the higher circulating MMP-3 concentrations observed in our cohort. However, these mechanisms were not directly evaluated in the present study; these interpretations remain speculative and requires further investigation.

Our previous work has shown that circulating MMP-3 is elevated in acute lung injury and acute respiratory distress syndrome (ARDS), and that higher plasma concentrations are associated with worse outcomes in critically ill patients. In this study, the biomarker analysis of the albuterol to treat acute lung injury (ALTA) trial, MMP-3 predicted 90-day mortality with good discrimination, supporting its relevance as a severity-linked marker in ARDS [12]. MMP-3 has also been suggested as a potential diagnostic and therapeutic target in severe COVID-19, given its link to early lung and endothelial injury and supported by experimental evidence showing that MMP-3 inhibition can reduce vascular permeability in preclinical models of ARDS [13]. Together, these findings indicate that MMP-3 consistently tracks with acute respiratory injury across different settings, and the present study extends this work by characterizing MMP-3 levels specifically in viral pneumonia.

Interestingly, higher MMP-3 concentrations were associated with higher PaO_2_/FiO_2_ ratio in COVID-19 cohort, implying a relationship with oxygenation status. This finding differs from the expected association between biomarkers of lung injury and worsening respiratory function. Additional studies are needed to determine whether this relationship reflects biological differences in disease pathophysiology or is attributable to cohort-specific factors.

An important finding in our study was the negative correlation between plasma MMP-3 and bicarbonate (HCO_3_^−^) levels, which was stronger and significant among patients with H1N1 compared with those with COVID-19. This observation may indicate a relationship between increased MMP-3 levels and systemic acid- base disturbances during severe viral pneumonia. Volkov et al. reported significantly lower bicarbonate concentrations in patients with H1N1 associated ARDS compared with those with COVID-19, suggesting differences in systemic physiological and acid-base responses between two viral infections [14].

This study has several strengths. We evaluated MMP-3 in two well-defined viral pneumonias, H1N1 and COVID-19, using standardized sampling and uniform assay procedures. The direct comparison between these two infections is uncommon in the existing literature, and our findings provide new evidence that circulating MMP-3 behaves differently across viral etiologies. Although correlations with most severity indices did not reach statistical significance in the H1N1 subgroup, higher MMP-3 levels were associated with lower bicarbonate concentrations, suggesting a potential relationship between MMP-3 and disease physiology in influenza-related pneumonia. However, certain limitations should be acknowledged. In our study, we did not recruit healthy donors as controls. The sample size was relatively small, which makes subgroup differences harder to interpret. The differences in treatment strategies between the two viral infections may also have influenced circulating MMP-3 concentrations and clinical outcomes. Moreover, some important clinical covariates were unavailable/missing to include in the current study, yet we analyzed using available data. Our findings have not been externally validated in an independent cohort and the prognostic value of MMP-3 requires confirmation using multivariable analyses to determine whether MMP-3 provides independent prognostic information beyond established clinical severity measures. In addition, we relied on previously collected data and could not evaluate changes in MMP-3 over time or determine cause-and-effect relationships. Lastly, we did not perform mechanistic experiments, so the biological reasons for the differences in MMP-3 levels between H1N1 and COVID-19 cannot be confirmed. Even with these limitations, the study adds useful comparative data on MMP-3 in viral pneumonia.

## 5. Conclusions

In conclusion, we found that circulating MMP-3 levels differed markedly between viral pneumonias, with significantly higher concentrations in H1N1 compared with COVID-19. In the overall cohort, higher MMP-3 levels were associated with worse clinical characteristics, although these findings may partly reflect differences between viral etiologies. These findings suggest that MMP-3 reflects aspects of acute disease burden in viral pneumonia and may help distinguish between different viral etiologies. While further studies with larger cohorts and mechanistic evaluation are needed, our results provide new comparative data on the behavior of MMP-3 in H1N1 and COVID-19 infections.

## Figures and Tables

**Table 1:** Demographic analysis of COVID-19 and H1N1.

Variable	COVID-19 (*n* = 52)	H1N1 (*n* = 57)	*p* Value
Age, years (mean ± SD)	50.7 ± 13.4	47.5 ± 10.8	0.176
Male sex, *n* (%)	24 (46.2)	31 (54.4)	0.505
Smoking history, *n* (%)	7 (13.5)	20 (35.1)	0.017
Length of stay, days	9.0 (6.0–20.0)	26.0 (16.2–33.0)	<0.001
Mechanical ventilation, *n* (%)	21 (40.4)	47 (82.5)	<0.001
Mortality, *n* (%)	8 (15.4)	11 (19.3)	0.775
Diabetes mellitus, *n* (%)	14 (26.9)	9 (15.8)	0.235
COPD, *n* (%)	1 (1.9)	0 (0.0)	0.477
Cancer, *n* (%)	0 (0.0)	0 (0.0)	1.000
Tuberculosis, *n* (%)	1 (1.9)	0 (0.0)	0.477
Obstructive sleep apnea, *n* (%)	0 (0.0)	1 (1.8)	1.000
Autoimmune disorders, *n* (%)	16 (30.8)	1 (1.8)	<0.001
Systemic arterial hypertension (SAH), *n* (%)	11 (21.2)	10 (17.5)	0.815
Time from symptom onset to blood sampling time (days)	8.0 (6.0–11.5)	8.0 (5.25–11.0)	0.636
MMP-3 (ng/mL), mean ± SD	3.98 ± 0.75	6.45 ± 1.35	<0.001

Statistical test showing the comparison between the COVID-19 and H1N1 groups. Data are presented as median (interquartile range) for data that were not normally distributed or mean  ±  SD for data that were normally distributed.

**Table 2:** Demographic analysis based on low MMP-3 and high MMP-3 levels.

Variable	Low MMP-3 (*n* = 49)	High MMP-3 (*n* = 60)	*p*-Value
COVID-19, *n* (%)	47 (95.9)	5 (8.3)	<0.001
H1N1, *n* (%)	2 (4.1)	55 (91.7)	<0.001
Age (years)	51.0 (42.0–58.0)	48.5 (41.8–57.0)	0.786
PaO_2_/FiO_2_ (*n* = 108)	188.0 (118.0–241.1)	101.5 (60.0–175.3)	<0.001
HCO_3_ (mmol/L)	22.4 (20.0–24.6)	22.5 (20.3–23.9)	0.798
SOFA score (*n* = 102)	2.0 (2.0–4.0)	6.0 (4.0–8.0)	<0.001
APACHE II score (*n* = 94)	6.5 (3.0–12.0)	9.0 (6.3–14.8)	0.010
Hospital length of stay (days)	11.0 (6.0–24.0)	24.0 (15.0–33.0)	0.001
Mortality, *n* (%)	8 (16.3)	11 (18.3)	0.806

Statistical test showing the comparison between low MMP-3 and high MMP-3 groups. Data are presented as median (interquartile range) for data that were not normally distributed or mean  ±  SD for data that were normally distributed. Definition of abbreviations: PaO_2_: Partial pressure of oxygen; FiO_2_: Fraction of inspired oxygen; APACHE II: Acute Physiology and Chronic Health Evaluation II; SOFA: Sequential Organ Failure Assessment score.

**Table 3:** Association of plasma MMP-3 with disease severity measures after adjustment for viral etiology.

Outcome	N	MMP-3 (β)	MMP-3 (*p*)	Etiology (*p*)
SOFA	102	−0.334	0.169	<0.001
APACHE II	94	−0.700	0.221	<0.001
Length of Stay	102	0.558	0.757	0.045
PaO_2_/FiO_2_	103	19.71	0.012	<0.001

β coefficients and corresponding *p* values were obtained from multivariable linear regression models evaluating the association between plasma MMP-3 concentrations and clinical severity parameters after adjustment for viral etiology. Viral etiology was included as a covariate in all regression models. Separate models were constructed for each clinical outcome. Statistical significance was defined as *p* < 0.05.

## Data Availability

Data will be made available on request from the corresponding author, D.Z.

## Abbreviations

The following abbreviations are used in this manuscript:

MMP-3	Matrix metalloproteinase-3
COVID-19	Coronavirus disease 2019
SARS-CoV-2	Severe acute respiratory syndrome coronavirus 2
H1N1	Influenza A/H1N1 virus
ELISA	Enzyme-linked immunosorbent assay
PaO_2_	Partial pressure of arterial oxygen
FiO_2_	Fraction of inspired oxygen
PaO_2_/FiO_2_	Ratio of arterial oxygen partial pressure to inspired oxygen fraction
SOFA	Sequential Organ Failure Assessment
APACHE II	Acute Physiology and Chronic Health Evaluation II
ARDS	Acute respiratory distress syndrome
AUROC	Area under the receiver operating characteristic curve
AUC	Area under the curve
WHO	World Health Organization
IL-1β	Interleukin-1 beta
IL-6	Interleukin-6
IRB	Institutional Review Board
RT-PCR	Reverse transcription polymerase chain reaction
SD	Standard deviation
IQR	Interquartile range
